# COVID-19 behavioral health and quality of life

**DOI:** 10.1038/s41598-022-05042-z

**Published:** 2022-01-19

**Authors:** Tonya Cross Hansel, Leia Y. Saltzman, Pamela A. Melton, Tanisha L. Clark, Patrick S. Bordnick

**Affiliations:** Tulane University, New Orleans, USA

**Keywords:** Risk factors, Signs and symptoms

## Abstract

In addition to concern about physical health consequences of COVID-19, many researchers also note the concerning impact on behavioral health and quality of life due to disruption. The purpose of this paper is to explore pathways of COVID-19 behavioral health and quality of life. We found increased anxiety, depression, and alcohol misuse and that the pandemic exacerbated prior problems. Further community indicators also lead to poorer behavioral health and overall decreased quality of life. The nature of COVID-19 and vast reach of the virus suggests that behavioral health concerns should take a primary role in pandemic recovery.

## Introduction

Decreased cases and death rates, along with increased vaccination coverage rates and lifted restrictions, are all markers that the COVID-19 pandemic is nearing the recovery phase of disaster response. While many are looking forward to their new normal or returning to pre-pandemic lifestyle, continued threat, vaccine uncertainties, and strain variation serve as cautionary reminders that the global pandemic is ongoing. Regardless of how long it takes for full recovery, more than a year of heightened fears, loneliness, economic consequences, and grief suggest that behavioral health will have longer term consequences^1^. In disaster mental health, when the threat has dissipated and individuals move out of survival mode, behavioral health problems become more apparent, and consequently services, such as psychoeducation, therapy and brief treatments, are needed^2,3^.

Limited research from previous pandemics suggest demographic indicators of poorer mental health, specifically for females, youth, and lower education levels^4,5^. Pandemic-related resource loss also contributed to mental health problems, including increased work, family, and financial stress coupled with decreased social support^4,6,7^. Direct exposure to the virus, including being a healthcare worker, being sick, or having sick family members, all had elevated symptomatology^8–11^. Whether it is a social determinant, exposure to SARs, H1N1, and Ebola help understand that pandemics can result in elevated and long lasting mental health symptoms^6,9,12^.

Early studies from the COVID-19 pandemic identify similar concerns to past pandemics regarding mental health^5,13–15^. Age appears to be a predictor of mental health problems—younger adults have more difficulty^16–19^. However, another study found that persons older than 85 years had worsened mental health, suggesting a potential U-shape distribution^20^. Female study participants also tend to report higher symptomatology^21–23^. Financial loss, lack of social interactions, and COVID-19 experiences also contribute to mental health concerns, which is consistent with past pandemics^21,23,24^. Overall studies point to increased mental health problems; yet there is limited information beyond demographics and COVID-19 disruption on factors that contribute to concerning outcomes.

### Substance use

One particular concern is the increase in unhealthy coping mechanisms, such as alcohol misuse. Long-term disasters like the current pandemic place individuals and communities at increased risk for exhausting positive coping skills and turning to negative ones such as alcohol misuse^25–27^. Numerous studies report increases in stress, anger, anxiety, and depression, resulting in substance abuse and relapse post disaster^28–31^. For example, data from the National Survey on Drug Use and Health from 2007–2011 indicate that alcohol and marijuana use increased post disaster^32^, which may have resulted from perceived or real loss of income, loss of confidence in authorities, and loss of one's culture or way of life^33^. Given these experiences are likely to occur during the pandemic^2,34^, there is an emergent need to understand the magnitude and impact of these negative factors on alcohol misuse from COVID-19.

### Physical health

Adverse physical health conditions (e.g. respiratory problems, headaches, musculoskeletal problems, and somatic complaints) are the most commonly reported symptomology among disaster survivors^35,36^. A study of Hurricane Ike survivors concluded that 45.1% of the study population exhibited functional impairment in performing essential daily living skills (e.g. maintaining social connectedness and academic and work-related responsibilities), 52.6% reported poor health (e.g. disability), and 74.9% experienced depression^37^. Similarly, the 2019 novel coronavirus disease (COVID-19) global pandemic is a disaster of unprecedented measure. Individuals have feared for self and family members' well-being, have been socially isolated from family and friends, and have endured interrupted, prolong, and unresolved grief^2,38,39^. Consequently, behavioral symptoms (e.g. depression, anxiety, and psychological distress) are increasingly prevalent among COVID-19 survivors, and behavioral health symptoms are also underdiagnosed, undertreated, and under researched^40^.

In addition to having an increased prevalence of behavioral health concerns, COVID-19 survivors have an increased prevalence of comorbid conditions that lead to worsened clinical outcomes^41^. Empirical evidence supports a bidirectional link between mental health and physical health, thereby increasing the risk of comorbid sequelae for disability, morbidity, and mortality, a frequent cause of emotional distress, and is associated with a diminished quality of life for COVID-19 survivors^19,42^.

### Quality of life

Quality of life and overall well-being are highly attributed resources available and can be a protective factor for mental health. In studies following flu epidemics, those recovering reported decreased quality of life^43,44^. Recent COVID-19 studies have shown the interconnection of quality of life and mental health problems for recovery^45^. Liu and colleagues^46^ found that individuals with prior mental health problems had poorer health-related quality of life and reported more COVID-19-related disturbances. An early study found an increase in positive well-being since COVID-19, but it may have been due to looking at their past rather than future perceptions^47^. Other studies have shown significant declines in subjective well-being^48^ and quality of life^49^. Studies have shown that well-being may be beyond the individuals and influenced more by socio-environmental and community factors, such as GDP, healthcare access, and pandemic communication and response^50,51^. Quality of life and well-being are important to foster and are of great concern for individuals and for overall community recovery.

In a systematic review of recent studies regarding COVID mental health, common risk factors associated with mental distress during the COVID-19 pandemic include female sex, 40 years of age or younger, presence of mental or physical illnesses, and financial loss or unemployment^12^. However, only one study included U.S. participants, and more studies are needed to better inform local recovery plans regarding additional protective factors. Behavioral health is important for overall individual well-being, and also plays a role in collective prevention and risk^52^. The purpose of this paper is to explore pathways of COVID-19 behavioral health and quality of life. We hypothesize behavioral health indicators will have increased from 2019 population estimates and from participant perceived problems prior to COVID-19.

## Method

Sample selection for this cross-sectional study included responses from April 7, 2020, through July 26, 2020, and respondents ages 18–65. Electronic recruitment was conducted through Tulane University School of Social Work website and media promotions requesting voluntary participation through a Qualtrics link. Participants gave virtual consent by continuing the online survey and were informed they could skip any questions or stop at any time—there was no compensation for time. Adults (18 years of age or older) and access to the technological platform were the only limiting factors. The SAMHSA national hotline was provided at the end of the survey to connect participants with resources or if they incurred distress. Tulane University Institutional Review Board approved study protocol and were performed in accordance with relevant guidelines and regulations.

### Measures

Demographics (age, race, marital status, and income) were collected, along with participants completed dichotomous pre-existing COVID items. Participants were asked if they experienced mental health, physical health, or alcohol problems prior to the COVID-19 pandemic. In addition, valid measures of behavioral health, quality of life, and COVID impact were used.

#### Behavioral health

Behavioral health was assessed by anxiety, depression and alcohol misuse. Anxiety was measured by the General Anxiety Disorder 2 item scale, which asks in the past 30 days, were participants bothered by 1) feeling nervous, anxious, or on edge; and 2) not being able to stop or control worrying^53^. The GAD2 cut point was 3 and 53% met the cut-off (*M* = 4.9, *SD* = 1.9; α = 0.88). Depression was measured with the 2-item Patient Health Questionnaire, which asks if, in the past 30 days, participants were bothered by 1) little interest or pleasure in doing things and 2) feeling down, depressed, or hopeless^54^. The PHQ2 cut point was 3 and 28% met the cut-off (*M* = 3.9, *SD* = 1.7; α = 0.86. Alcohol misuse was measured by the CAGE, which asks participants if, in the past 30 days, they have: 1) felt you should **C**ut down on drinking; 2) been **A**nnoyed when people have commented on drinking; 3) felt **G**uilty or badly about your drinking; 3) had an **E**ye opener first thing in the morning to steady your nerves or get rid of a hangover^55^. The CAGE cut point was 1 and 14% met the cut-off; (*M* = 0.4, *SD* = 0.9; κ = 0.66.

#### Quality of life

Overall quality of life was measured with items selected from the World Health Organization WHOQOL-BREF quality of life assessment^56^. Participants were asked how good or satisfied they have felt over the last 2 weeks with their: quality of life, health, sleep, performance of daily living activities, capacity for work, conditions of your living place, and access to health services (*M* = 25.1, *SD* = 4.9; α = 0.74).

#### COVID-19

Impact was assessed through COVID experiences and COVID disruption. Participants were asked to respond to whether they had experienced the following as a result of COVID 19: loss of usual way of life, social isolation, work from home, children and adolescents being out of school, loss of income or revenue, personal health effects, participated in response or emergency services, and COVID-19 suspected or diagnosed, loss of job or business, COVID-19 diagnosis. A COVID experience index was created where 1 point was given for experiences listed. Items for the COVID disruption were adapted from the Sheena Disability Scales^57^. Participants were also asked to what degree the pandemic had disrupted their work/school life, social/leisure activities, and family/home responsibilities activities (*M* = 11.6, *SD* = 2.4; α = 0.61).

#### Community behavioral health

County-level data was accessed from the Robert Wood Johnson Better Health Data^58^. Data were matched to participants’ zip codes and included: percentage of excessive drinking, average number of mentally unhealthy days, and average number of physically unhealthy days. County-level COVID death and diagnosis rates through October 2020 were also accessed from the Center for Disease Control COVID Data Tracker^59^.

### Participants

The participants (*N* = 296) represented many states, including Louisiana (55%), Texas (6%), California (5%), Florida (3%), Georgia (3%), Illinois (3%); 2% percent from Mississippi, Pennsylvania, Virginia, North Carolina, and Massachusetts; and 1% from New Jersey, New York, Arizona, Iowa, Maryland, Michigan, Missouri, Connecticut, South Carolina, Kentucky, Ohio, Minnesota, Oregon, Washington; and less than 1% (0.3%) representation from Rhode Island, Delaware, Alabama, Tennessee, Indiana, Wisconsin, Nebraska, Colorado, Wyoming, Alaska.

The minimum age was 19 and the maximum was 65 (*M* = 43.6, *SD* = 12.5); 85% identified as women, 14% as men, and 1% as nonbinary. Participants were allowed to select multiple racial/ethnic identities, the majority identified as White (86%); 8% identified Black or African American, 6% were Latinx, Latin@ or Hispanic, 3% identified as Asian, and 1% identified as Native American or Alaskan Native. The median 2019 income was $60,000– $69,999. All participants had at least a high school education (18%) and 72% had a 4-year or professional degree, 10% had a doctorate. The majority of participants (65%) were married or cohabitating; 26% were single, 8% were divorced or separated, and 1% were widowed.

### Data analysis

Data analyses were conducted using Statistical Package for the Social Sciences (SPSS) version 27. Point biserial correlations were conducted among COVID-19 experiences and behavioral health variables. McNamara Chi square analyses were conducted to compare current cut-off scores (meeting cutoff for either anxiety or depression) or alcohol misuse cutoff with previous mental health and substance use problems. One sample *Z* tests were used to compare participant cut-off scores (anxiety, depression, and alcohol misuse) with 2019 population estimates. Zero order (Pearson product moment) correlations were conducted to assess associations among variables. The structural model was tested using SPSS analysis of moment structure (AMOS) version 27. Assumptions of normality and linearity were met; missing data was less than 5% and imputed using linear interpolation. Significant zero-order correlation paths were added to the model but did not reveal good fit (RMSE > 0.05). After reviewing regression weights, the dichotomized married versus nonmarried and minority versus nonminority were removed due to their lack of contribution to the model.

## Results

Prior behavioral health concerns were asked and 30% noted physical (*n* = 88) health problems, 29% mental health problems, and 4% substance use problems. Results suggest an increase in current mental health concerns (33%), compared to preexisting problems (25%), Χ^2^ (1) = 37.61, *p* < 0.001. Current alcohol use (12%) was also increased over previous substance use (2%), *Χ*^2^ (1) = 16.42, *p* < 0.001. Increased levels of anxiety 53% compared to 16% for 2019 population estimates ^60^, *Z* (296) = 17.4, *p* < 0.001. Increased levels of depression 28% for moderate compared to 19% for the population estimates^61^, *Z* (296) = 4.4, *p* < 0.001. Alcohol misuse (14%) was also increased compared to 2019 population estimates (6%) of heavy alcohol use^62^, *Z* (296) = 5.6, *p* < 0.001.

Participants were asked to report on COVID-19 experiences. Over one third of respondents reported COVID-19 experiences as social isolation, working from home, loss of income, and children and adolescents being out of school (see Table 1). Participants noting social isolation and personal health effects had higher anxiety and depression and lower quality of life. Participants with suspected or diagnosed COVID-19 reported more alcohol misuse and lower quality of life (see Table 1).

**Table 1:** Frequency, Percentages and Correlation Coefficients on COVID-19 Experiences.

	Frequency	Percent	Point biserial Correlation Coefficients			
			Anxiety	Depression	Alcohol Misuse	Quality of Life
Social isolation	219	74%	.21**	.26**	−.10	−.22**
Work from home	181	61%	.09	.05	.09	−.01
Children and adolescents being out of school	104	35%	−.02	−.03	.04	−.02
Loss of income	103	35%	.06	.05	−.01	−.13*
Personal health effects	42	14%	.26**	.23**	.07	−.33**
Participated in response or emergency services	46	15%	.03	−.03	.07	−.01
COVID-19 suspected or diagnosed	32	11%	.01	.04	.15*	−.12*

Significant values are in bold.

Zero order correlations are presented in Table 2. COVID-19 experiences and disruption were associated with increases in anxiety and depression and decreased quality of life. Younger participants reported more COVID-19 experiences and prior mental health concerns. Anxiety and depression were positively associated, and both were negatively associated with quality of life, suggesting that as depression and anxiety increase, quality of life decreases. Current anxiety and depressive symptoms were associated with prior physical and mental health problems. Females and whites reported more anxiety, while higher income was associated with higher quality of life scores. Respondents with higher anxiety scores lived in communities with a higher number of physically unhealthy days.

**Table 2:** Zero Order Coefficients and Descriptive Statistics.

		M	SD	1	2	3	4	5	6	7	8	9	10	11	12	13	14	15	16	17
1	Anxiety	4.9	1.9	1																
2	Depression	3.9	1.7	.62**	1															
3	Alcohol misuse	0.4	0.9	.04	.12*	1														
4	Quality of life	25.1	4.9	−.38**	−.45**	−.05	1													
5	% Excessive Drinking	20.4	2.2	.07	.01	.08	−.01	1												
6	# Mentally Unhealthy Days	4.4	0.6	.13*	.06	.06	−.04	.20**	1											
7	# Physically Unhealthy Days	4.1	0.6	.12*	.09	.04	−.03	.15*	.94**	1										
8	Age	43.6	12.6	−.09	−.09	−.08	.04	−.01	−.104	−.14*	1									
9	2019 income	7.2	3.6	−.05	−.14*	−.07	.21**	−.01	−.21**	−.24**	.43**	1								
10	Minority	0.8	0.4	.13*	−.01	−.09	−.01	.08	−.01	−.02	.18**	.14*	1							
11	Gender	0.9	0.4	.18**	.10	−.01	−.04	−.02	−.01	−.01	−.01	−.02	.17**	1						
12	Married	0.7	0.5	−.05	−.08	.05	.09	.01	−.03	−.05	.32**	.34**	.08	−.01	1					
13	Pre physical health problems	0.3	0.5	.14*	.12*	−.17**	−.16**	−.13*	.01	−.01	.16**	.08	.01	.04	−.01	1				
14	Pre Mental health problems	0.3	0.5	.30**	.31**	.02	−.25**	.08	.03	.08	−.37**	−.25**	.04	.02	−.11*	.07	1			
15	Pre alcohol problems	0.0	0.2	.01	.09	.29**	−.03	.01	−.04	−.04	−.11	.01	−.11	−.11	−.17**	.02	.21**	1		
16	COVID-19 rate	12,201.3	5041.7	.01	.05	.17**	−.03	.21**	.36**	.24**	−.04	−.08	−.09	−.05	−.01	−.08	−.01	−.02	1	
17	COVID-19 experiences	2.5	1.2	.22**	.19**	.09	−.27**	.04	−.02	−.02	−.11*	−.02	−.01	.06	.08	.05	.22**	.02	.03	1
18	COVID-19 disruption	11.6	2.4	.31**	.28**	.02	−.29**	−.01	−.01	−.04	−.01	−.07	.04	.01	.05	−.09	.03	−.02	.01	.40**

Significant values are in bold.

*p < .05, **p < .001.

Respondents who reported prior physical health problems were older, had more alcohol misuse, had decreased quality of life, and were less likely to live in communities with excessive drinking. Respondents who reported prior mental health problems had lower quality of life, lower incomes, and were younger. As current alcohol misuse increased, depression increased, and respondents also tended to live in communities with higher COVID-19 rates. Individuals reporting prior substance use problems had higher alcohol misuse scores, were less likely to be married, and reported prior mental health problems. Communities with more physically unhealthy days also had increased percentages of excessive drinking. Respondents who were older lived in communities with higher numbers of physically unhealthy days. Respondents with lower 2019 income reported higher depression. They were more likely to live in communities with more physically and mentally unhealthy days. Communities with higher COVID-19 rates also had higher averages of mentally and physically unhealthy days and percentages of excessive drinking.

### Structural model

**Figure 1:**
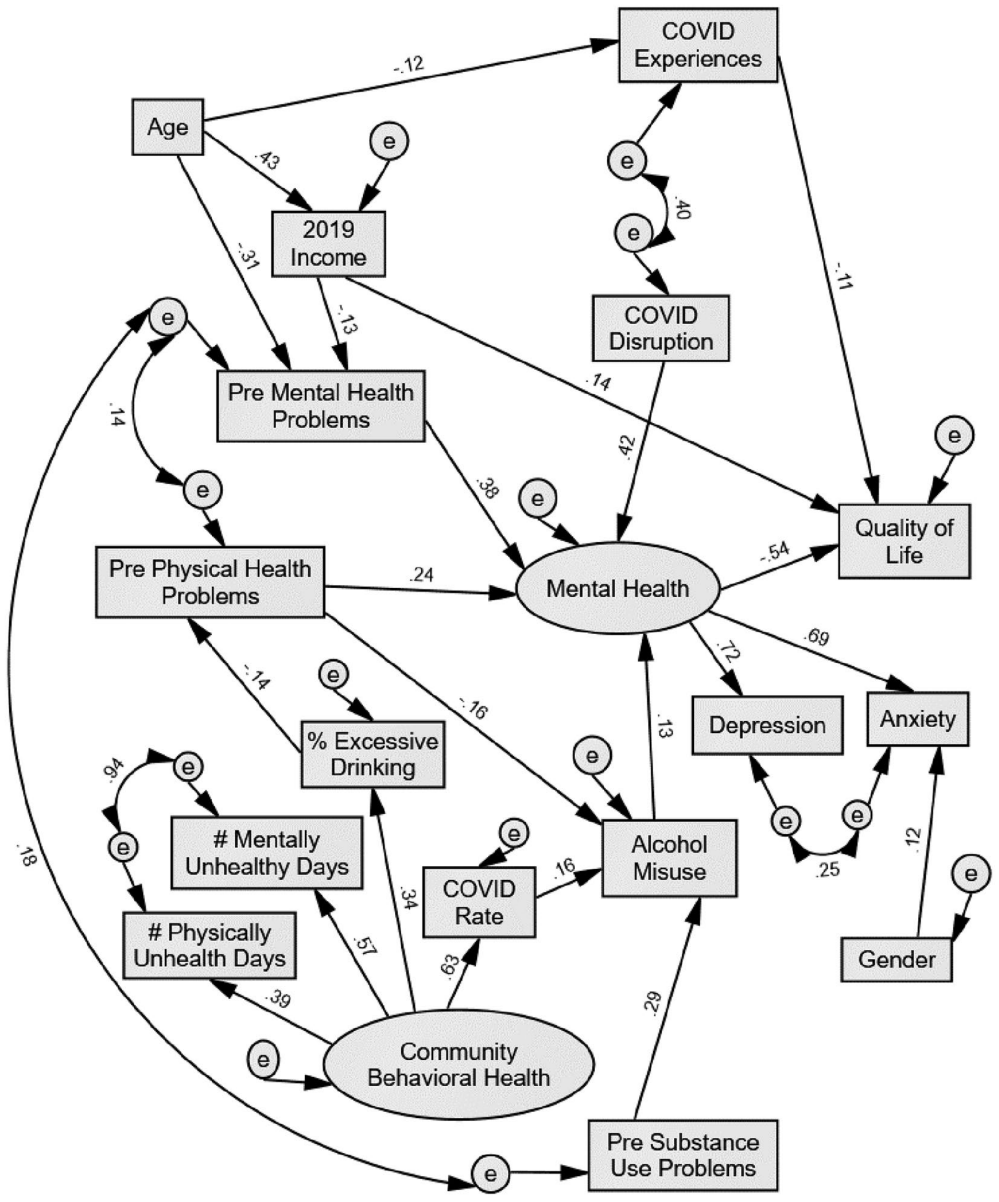
COVID-19 Behavioral Health Model.

The final model was acceptable given the smaller sample size and use of dichotomous pre COVID-19 behavioral health variables, χ2 (93) = 123.7, *p* = 0.018, CFI = 0.976, RMSEA = 0.033 (lower 0.15, upper 0.48); PNFI of 0.623, suggests the overall model accounts for approximately 62% of the variance in behavioral health and quality of life. Table 3 presents the path coefficients and model estimates. The largest contributors to the model are as follows: mental (β = 0.39, *p* < 0.001) and physical health (β = 0.24, *p* < 0.001) problems prior to COVID-19 were predictive of current mental health (latent variable including anxiety and depression). Mental health was predictive of quality of life (β = -0.55, *p* < 0.001). Younger age was predictive of prior mental health problems (β = -0.31, *p* < 0.001). Prior substance use problems were predictive of current alcohol misuse (β = 0.29, *p* < 0.001) and COVID-19 disruption was predictive of mental health (β = 0.42, *p* < 0.001). Figure 1 presents the final model, where significant standardized estimates are shown next to each path, and the coefficient for each variable’s contribution to the model is also included.

**Table 3:** Path Coefficients.

			**Estimate**	**SE**	**CR**	***p***	**Standardized Estimates**
% Excessive Drinking	←	Community behavioral health	3.47	1.14	3.04	.002	0.34
Pre physical health problems	←	% Excessive Drinking	−0.03	0.01	−2.50	.012	−0.14
Income	←	Age	0.12	0.02	8.10	***	0.43
COVID-19 rate	←	Community behavioral health	14,612.88	4837.86	3.02	.003	0.63
Pre Mental health problems	←	Income	−0.02	0.01	−2.13	.033	−0.13
Pre Mental health problems	←	Age	−0.01	0.00	−5.30	***	−0.31
Alcohol misuse	←	Pre substance use problems	1.29	0.24	5.40	***	0.29
Alcohol misuse	←	Pre physical health problems	−0.30	0.10	−2.91	.004	−0.16
Alcohol misuse	←	COVID-19 rate	0.00	0.00	2.92	.003	0.16
Mental Health	←	Pre physical health problems	0.70	0.18	3.83	***	0.24
COVID-19 Experience	←	Age	−0.01	0.01	−2.18	.029	−0.12
Mental Health	←	COVID-19 disruption	0.23	0.04	6.41	***	0.42
Mental Health	←	Pre Mental health problems	1.12	0.19	5.87	***	0.39
Mental Health	←	Alcohol misuse	0.19	0.09	2.04	.041	0.13
Quality of Life	←	COVID-19 Experiences	−0.46	0.21	−2.21	.027	−0.11
Quality of Life	←	Mental Health	−2.02	0.33	−6.04	***	−0.55
# Physically Unhealthy Days	←	Community behavioral health	1.00				0.39
# Mentally Unhealthy Days	←	Community behavioral health	1.49	0.14	10.62	***	0.57
Anxiety	←	Gender	0.64	0.24	2.72	.007	0.12
Anxiety	←	Mental Health	1.00				0.69
Quality of Life	←	Income	0.19	0.07	2.81	.005	0.14
Depression	←	Mental Health	0.94	0.09	10.31	***	0.72

***p < .001.

## Discussion

Increased anxiety during a pandemic has led to preventative behaviors, such as increased handwashing and following safety restrictions, demonstrating a protective factor of mild anxiety^52^. However, ongoing stressors become cumulative and can lead to longer-term mental health problems^64^. Biological disasters like the COVID-19 pandemic create a large amount of disruption, uncertainty, and public fear—globally impacting communities for over a year. There is a significant gap in the literature regarding behavioral health and quality of life during global pandemics. Overall results of this study suggest complex pathways to COVID-19 behavioral health and subsequent quality of life.

### Behavioral health

The current study revealed that COVID-19 disruption to participants’ work, family, or social life uniquely contributed to poorer mental health. Specifically, participants noting social isolation and personal health effects exhibited worsened mental health. These findings are in line with existing research on pandemic contributors to mental health concerns, including loneliness^1^ and direct exposure^10^. As one study noted, increased experiences of COVID-19 at the height of the pandemic produced occurrences of psychological distress that created or increased mental illness in China^64^. Our study found that COVID-19 experiences likely exacerbated existing problems, where prior mental and physical health were predictive of current anxious and depressive symptoms. Similarly, Young and colleagues^65^ found those who experienced mental health symptoms in the past were at an increased risk for severe mental health diagnoses. Further, early studies and our results support the growing literature of concerns for longer-term mental health problems due to COVID-19^17^.

In a systematic review of recent literature surrounding COVID-19 Xiong and colleagues^12^, found that increases in depression, anxiety, and PTSD symptoms were associated with certain risk factors. Similarly, our study found indirect pathways to mental health, where younger participants reported prior mental health problems and older participants reported prior physical health problems. In the current study, younger participants also had more indirect stressors associated with the pandemic^16^ and reported more COVID-19 experiences. Females and whites reported more anxiety, although this association did not hold in the structural model. Similar results have been found in other studies regarding females^20^, but the opposite regarding race and ethnicity^22,66^. Discrepancies are likely due to the significant association among older participants identifying as members of a minority grouping.

Another contributor to poor behavioral health is substance use. Early COVID-19 studies estimate over 10% started or increased substance misuse as a coping mechanism^67^. Our study supports the increase of substance due to COVID-19, where current alcohol misuse was associated with prior substance use. Further, prior substance use was associated with prior mental health problems; this connection was continued for current usage, where alcohol misuse was also associated with depression. Following the Ebola outbreak, one risk factor identified was increased substance use, which can exacerbate negative mental health outcomes^68^. In the current study, participants with suspected or diagnosed COVID-19 or who lived in communities with higher rates reported higher alcohol misuse, suggesting use of alcohol as a potential negative coping mechanism.

### Quality of life

Most would expect quality of life to be challenged during a global pandemic; however, when we assessed behavioral health as a component of overall quality of life, longer term outcomes became concerning. Both past and current mental health were strong predictors of quality of life. Other studies have demonstrated this connection for individuals recovering from COVID-19^45,46,69^. COVID-19 experiences play a large role in quality of life, where participants who felt socially isolated reported personal health effects, or COVID-19 suspected or diagnosed participants reported lower quality of life. Similar to a large international study by Alzueta and colleagues^13^, the number of experiences related to COVID-19 played a role in overall well-being. Specifically, we found that increased COVID-19 experiences predicted lower quality of life, demonstrating an allostatic load effect and the accumulation of pandemic-related stressors toward negative health outcomes^63^. Individuals who do not adapt well to taxing life events tended to experience increased rates of anxiety and depression, which lowers quality of life.

### Place matters

Place matters with regards to COVID-19, where behavioral health and community factors contributed to overall health. A community consists of individuals one can identify with. Specifically, the community is place-based or labeled as a locality where individuals who comprise the community interact to share social capital. The social capital theory contends that social connectedness is a resource that guides the growth and accumulation of interpersonal relationships^70^. Community-based findings from this study found that participants with higher anxiety scores and who were older lived in communities with more physically unhealthy days. Respondents with lower 2019 income were less likely to live in communities with more physically and mentally unhealthy days. Respondents who reported prior physical health were less likely to live in communities with excessive drinking. Yet communities with overall poorer health also had increased percentages of excessive drinking. Communities with higher COVID-19 rates also had higher averages of mentally and physically unhealthy days and percentages of excessive drinking. Similar results demonstrated that higher individual and community stressors result in poor mental health and inadvertently decrease quality of life^12,71,72^. Communities' social capital works as a protective factor against the accumulative effects of COVID-19 (e.g. social isolation, depression, psychological distress, and deaths)^73,74^.

## Limitations and future research

Future studies are needed to understand protective factors (e.g. self-care, technology-aided connectedness) that can buffer more negative effects. Researchers from Turkey observed a reduction in COVID-19 anxiety and depression symptomology following physical activity programs to increase optimal health functioning, social connectedness, decreased anxiety and depression symptoms^75^. Similar programs and studies are needed to understand buffering effects on U.S. populations. Respondents were largely female (85%) and while consistent with existing studies that more females respond to surveys^76^, this may impact results. Timing is a major consideration for this study, as behavioral health needs and concerns are likely to change over the course of the pandemic^2^. Future studies are also needed to understand longer term behavioral health implications, social media effects, and family impacts, including parenting, adult caregiving, and youth^77^.

## Summary and impact

The nature of COVID-19 and vast reach of the virus suggest that behavioral health concerns should take a primary role in pandemic recovery. While we can expect many individuals with elevated symptoms or substance use problems to remit over time, the ongoing nature of the current pandemic is likely to yield longer-term reactions^5,6,78,79^. The continued direct and indirect effects of the pandemic alludes to the pandemic hindering improvements in people’s health and overall well-being. This study supports the urgent need for enhanced behavioral health service capacity moving into the recovery phase of the pandemic^80^. Based on past disasters, brief services such as Skills for Psychological Recovery^81^ are still needed to normalize mental health symptoms and awareness of risk factors and acknowledge problematic coping, such as alcohol use. Brief interventions may be necessary to boost coping skills that may be diminished due to COVID-19. At this point in the disaster, more intensive treatments should also be made available^82^, especially for those who exhibit specific risk factors, such as young and middle-aged adults, those with limited income and prior behavioral health concerns, and those living in communities with poorer health. Perhaps some of the gains made toward telehealth over the past year^83^ can continue and increase access and capacity to support improved behavioral health and quality of life.
